# Mapping of functional elements of the *Fab-6* boundary involved in the regulation of the *Abd-B* hox gene in *Drosophilamelanogaster*

**DOI:** 10.1038/s41598-021-83734-8

**Published:** 2021-02-18

**Authors:** Nikolay Postika, Paul Schedl, Pavel Georgiev, Olga Kyrchanova

**Affiliations:** 1 Department of the Control of Genetic Processes, Institute of Gene Biology Russian Academy of Sciences, 34/5 Vavilov St., Moscow, Russia 119334; 2 Laboratory of Gene Expression Regulation in Development, Institute of Gene Biology Russian Academy of Sciences, 34/5 Vavilov St., Moscow, Russia 119334; 3 Department of Molecular Biology, Princeton University, Princeton, NJ 08544 USA; 4 Center for Precision Genome Editing and Genetic Technologies for Biomedicine, Institute of Gene Biology Russian Academy of Sciences, 34/5 Vavilov St., Moscow, Russia 119334

**Keywords:** Genetics, Chromatin, Transcription

## Abstract

The autonomy of segment-specific regulatory domains in the *Bithorax* complex is conferred by boundary elements and associated Polycomb response elements (PREs). The *Fab-6* boundary is located at the junction of the *iab-5* and *iab-6* domains. Previous studies mapped it to a nuclease hypersensitive region 1 (HS1), while the *iab-6* PRE was mapped to a second hypersensitive region HS2 nearly 3 kb away. To analyze the role of HS1 and HS2 in boundary we generated deletions of HS1 or HS1 + HS2 that have *attP* site for boundary replacement experiments. The 1389 bp HS1 deletion can be rescued by a 529 bp core *Fab-6* sequence that includes two CTCF sites. However, *Fab-6* HS1 cannot rescue the HS1 + HS2 deletion or substitute for another BX-C boundary – *Fab-7*. For this it must be combined with a PRE, either *Fab-7* HS3, or *Fab-6* HS2. These findings suggest that the boundary function of *Fab-6* HS1 must be bolstered by a second element that has PRE activity.

## Introduction

**Figure 1:**
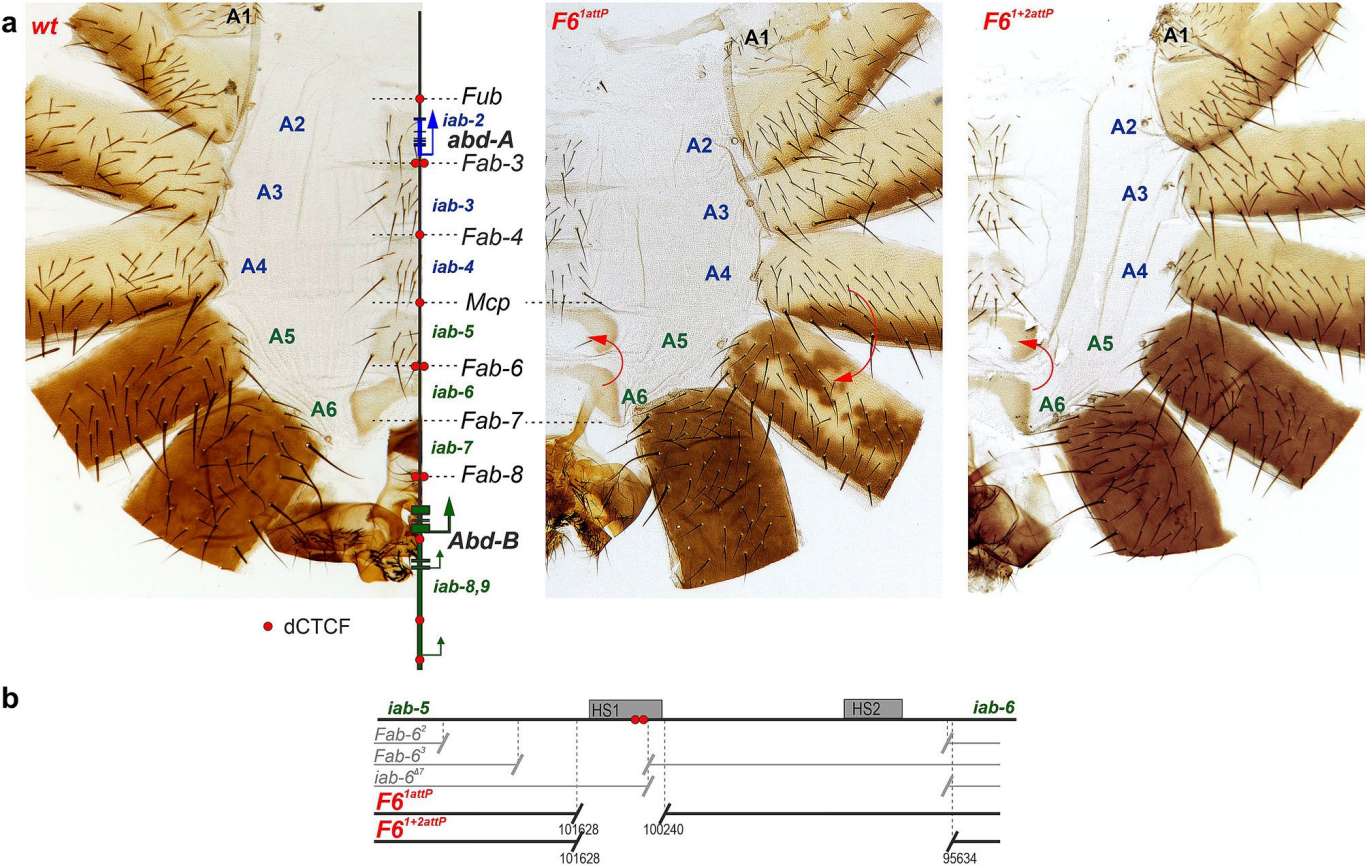
Deletions in the region of the *Fab-6* boundary. (**a**) Morphology of the male abdominal segments in *wild* *type* (*wt*), *F6*^*1attP*^ and *F6*^*1*+*2attP*^ alleles. The organization of the *abd-A* and *Abd-B* regulatory regions in *Bithorax* complex is shown near *wt* abdominal segments. The *abd-A* (blue) and *Abd-B* (green) promoters are schematically demonstrated by arrows. The abdominal segments and *iab*-domains that provide their features are shown (more in the text). A7 and A8 are absent in *wt* adult males. A9 is a part of male genitals. The lines with colored circles mark characterized (*Fub*, *Mcp*, *Fab-6*, *Fab-7*, and *Fab-8*) and predicted (*Fab-3*, and *Fab-4*) boundaries. The red circles indicate number of the CTCF binding sites in each boundary. *F6*^*1attP*^ and *F6*^*1*+*2attP*^: the red arc arrows show the direction of segment transformation. (**b**) Schematic representation of deletions mapped in the region of the *Fab-6* boundary. DNAse I hypersensitive sites (HS1 and HS2) are shown as gray boxes. Previously described deletions of the *Fab-6* boundary^8^ are indicated by breaks in the gray lines. The endpoints of *F6*^*1attP*^ and *F6*^*1*+*2attP*^ deletions used in the replacement experiments are indicated by breaks in the black lines. The coordinates of endpoints are according to the complete sequence of BX-C in SEQ89E numbering^61^.

The Drosophila *Bithorax* complex (BX-C) has three homeotic genes, *Ultrabithorax* (*Ubx*), *abdominal-A* (*abd-A*) and *Abdominal-B* (*Abd-B*), the pattern of expression of which in the posterior parasegments/segments, PS5-14/T3-A9 determines their identity^1–3^. This expression pattern is generated by a unique collection of tissue specific enhancers that are located in nine functionally autonomous *cis-*regulatory domains. Two of these domains, *abx/bx* and *bxd/pbx*, direct *Ubx* expression in PS5 (T3) and PS6 (A1). Three domains, *iab-2*, *iab-3* and *iab-4* control *abd-A* in PS7-9 (A2-4), respectively. Finally *Abd-B* expression in PS10-13 (A5-9) is controlled by four regulatory domains, *iab-5*, *iab-6*, *iab-7* and *iab-8,9* respectively^3,4^ (Fig. 1a).

BX-C regulation is divided into two phases, initiation and maintenance. During the initiation phase, which takes place around the blastoderm stage, a combination of gap, pair-rule gene and maternally derived transcription factors establishes parasegment identity by interacting with special initiator elements in each regulatory domain^3,5,6^. This interaction sets the activity state, either *on* or *off*, of the regulatory domains^3,7,8^. The BX-C regulatory domains are activated sequentially along the anterior–posterior axis. For example, *iab-5* is activated in PS10 and it directs *Abd-B* expression in this parasegment, while the remaining *Abd-B* regulatory domains *iab-6* - *iab-8,9* are *off*. In PS11, both *iab-5* and *iab-6* are activated; however, *iab-6*, which is closer to the *Abd-B* promoter, is responsible for regulating transcription. Again, the *iab-7* and *iab-8*,9 are *off*.

Once the gap, pair-rule and maternal gene products disappear during gastrulation, regulation of BX-C switches to the maintenance phase. In this phase the *on* and *off* states of the regulatory domains are maintained by Trithorax (Trx) and Polycomb (PcG) group proteins, respectively^3,9^. Trx and PcG family proteins are highly conserved and regulate enhancer and promoter activity mainly by introducing or removing histone modifications^10^. Special elements, called PREs (Polycomb Response Element) are responsible for recruiting these maintenance factors^11,12^. Originally discovered in BX-C, PREs were subsequently found to control many other developmental genes^10^. PREs in flies map to large nucleosome free regions and have sites for a complex array of DNA binding protein^11,12^. Included in this group are recognition motifs for the GAGA transcription factor, GAF, and the one known PcG protein that binds DNA, Pleiohomeotic (Pho)^13,14^. These two proteins cooperate in the recruitment of other PcG factors^15–19^, which then mark the surrounding nucleosomes with the H3K27me3. In BX-C this histone mark helps establish and maintain the *cis-*regulatory domains in the *off* state^20,21^. When the *cis*-regulatory domains are in the *on* state, PcG proteins and the H3K27me3 mark are replaced by Trx proteins and marks of active chromatin^22^.

Critical to the autonomous activity of the BX-C regulatory domains are another class of elements, called boundaries or insulators^23,24^. Each of the BX-C regulatory domains is separated from the adjacent domains by boundaries. For example, *iab-5* and *iab-6* are separated from each other by *Fab-6*, while *iab-6* is separated from *iab-7* by the *Fab-7* (Fig. 1). With the exception of *Mcp*, the boundaries in the *Abd-B* region (*Fab-6*, *Fab-7* and *Fab-8*) have two critical activities^25–32^. The first is blocking crosstalk between adjacent domains. Boundary deletions fuse flanking regulatory domains, allowing adventitious interactions between initiators (and PREs) in each domain. Typically, the initiator in the more proximal domain activates the fused domain leading to a gain-of-function (GOF) transformation. When *Fab-7* is deleted, for example, the *iab-6* initiator activates *iab-7* in PS11 where it would normally be *off*. This results in a transformation of PS11(A6) into PS12 (A7)^28^. Smaller *Fab-7* deletions that remove most of the boundary but retain *iab-7* PRE (HS3) give a mixed GOF and LOF (Loss-of-function) transformations^33^. In this case, the PRE is thought to silence some of the *iab-6,* *iab-7* enhancers. The second function is boundary bypass. This activity is required when there are boundaries between the regulatory domain and its target promoter. As illustrated in Fig. 1a, there are two boundaries, *Fab-7* and *Fab-8*, between *iab-6* and *Abd-B*. In order to drive *Abd-B* expression in PS11, the tissue specific enhancers in *iab-6* must bypass these two boundaries. Studies on *Fab-7* and *Fab-8* indicate that bypass activity, like insulation, is an intrinsic property^25,26,34^. However, not all BX-C boundaries have bypass activity, nor do they need this activity. For example, *Mcp* marks the border between the *abd-A* and *Abd-B* regulatory domains. In this location, bypass activity is not needed, and *Mcp* lacks this function^26^.

The most thoroughly studied BX-C boundary, *Fab-7*, consists of four hypersensitive regions, HS*, HS1, HS2 and HS3^35^. Transgene and endogenous replacement experiments indicate that it is composed of multiple, partially redundant elements. For example, in transgene assays, fragments spanning HS* + HS1 + HS2, have enhancer blocking activity^36,37^. These same sequences are sufficient to confer nearly full boundary function in the context of BX-C: they block *iab-6:iab-7* crosstalk and support bypass^27,38,39^. In transgene assays^14,16^ and also in genetic experiments^33^, *Fab-7* HS3 functions as a PRE. However, recent experiments showed that in addition to PRE activity, HS3 also has boundary function^27,39^. In fact, a combination of the distal half of HS1, dHS1, plus HS3, has full function in *Fab-7* boundary replacement experiments. Interestingly, a similar configuration of a boundary element and adjacent centromere distal PRE is observed for *Mcp*, *Fab-6* and *Fab-8,* suggesting that this organization may have some functional significance. On the other hand, for *Fab-8*, the available evidence indicates that the nearby PREs is not important for full boundary function^40^.

Here we have used boundary replacement experiments to analyze the functional properties of the *Fab-6* boundary. The chromosomal segment that is thought to contain *Fab-6* has two DNase I hypersensitive regions, HS1 and HS2 (Fig. 1b). Unlike other boundaries, both hypersensitive regions can function as PREs and silence *mini-white* reporters in transgene assays^41,42^. In addition, sequences spanning HS1 region also function as a boundary, blocking the *white* enhancer from activating *white*^41^. Consistent with this observation, there are two binding sites for the chromosome architectural protein dCTCF in HS1 and dCTCF together with CP190 are associated with HS1 *in* *vivo*^43,44^. Moreover, as might be expected, blocking activity of HS1 is partially compromised in *dCTCF* mutants^41^. Several other lines of evidence argue that HS1 likely corresponds to *Fab-6*. First, Iampietro et al.^8^ found that like other BX-C boundaries, deletion of HS1 results in a mixed GOF/LOF transformation of A5 towards A6 or A4. Second, Kyrchanova et al.^45^ showed that a 425 bp sequence spanning *Fab-6* HS1 functionally interacts with *Fab-7*, *Fab-8* and the promoter region of *Abd-B* in a transgene pairing assay. Here we report that the core of *Fab-6* boundary maps to a 529 bp sequence spanning HS1. While this core sequence can rescue a deletion spanning HS1, it cannot by itself rescue an HS1 + HS2 deletion, nor can it substitute for *Fab-7*. Instead HS1 must be combined with an HS2 fragment or the *Fab-7* HS3. Our studies indicate that *Fab-6* has two unusual features. One is the very large size of the sequences required for boundary function: two different DNA fragments of ~ 1 kb each are needed for boundary activity in the context of BX-C. The other is that both of these sequences not only have insulating activity but also function as PcG dependent silencers.

## Results

### The role of the *Fab-6* HS1 in the functioning of the *iab-5* and *iab-6* regulatory domains

Iampietro et al.^8^ generated several deletions in the region between *iab-5* and *iab-6* that were expected to remove sequences critical for boundary function. One of these, *Fab-6*^*2*^, removes ~ 8 kb including both HS1 and HS2. A second ~ 2 kb deletion, *Fab-6*^*3*^, extends from a site in *iab-5* ~ 1.1 kb from HS1 to a site within HS1 located just beyond the CTCF sites (Fig. 1b). Both deletions give a mixture of GOF/LOF phenotypes in A5 (PS10) and A6 (PS11). A third ~ 4,7 kb *iab-6*^*Δ7*^ deletion, that removes HS2, had no apparent phenotypic effects^8^. Based on these findings and those of Perez-Lluch et al.^41^, it was proposed that HS1 is the core *Fab-6* boundary while HS2 corresponds to the *iab-6* PRE.

**Figure 2:**
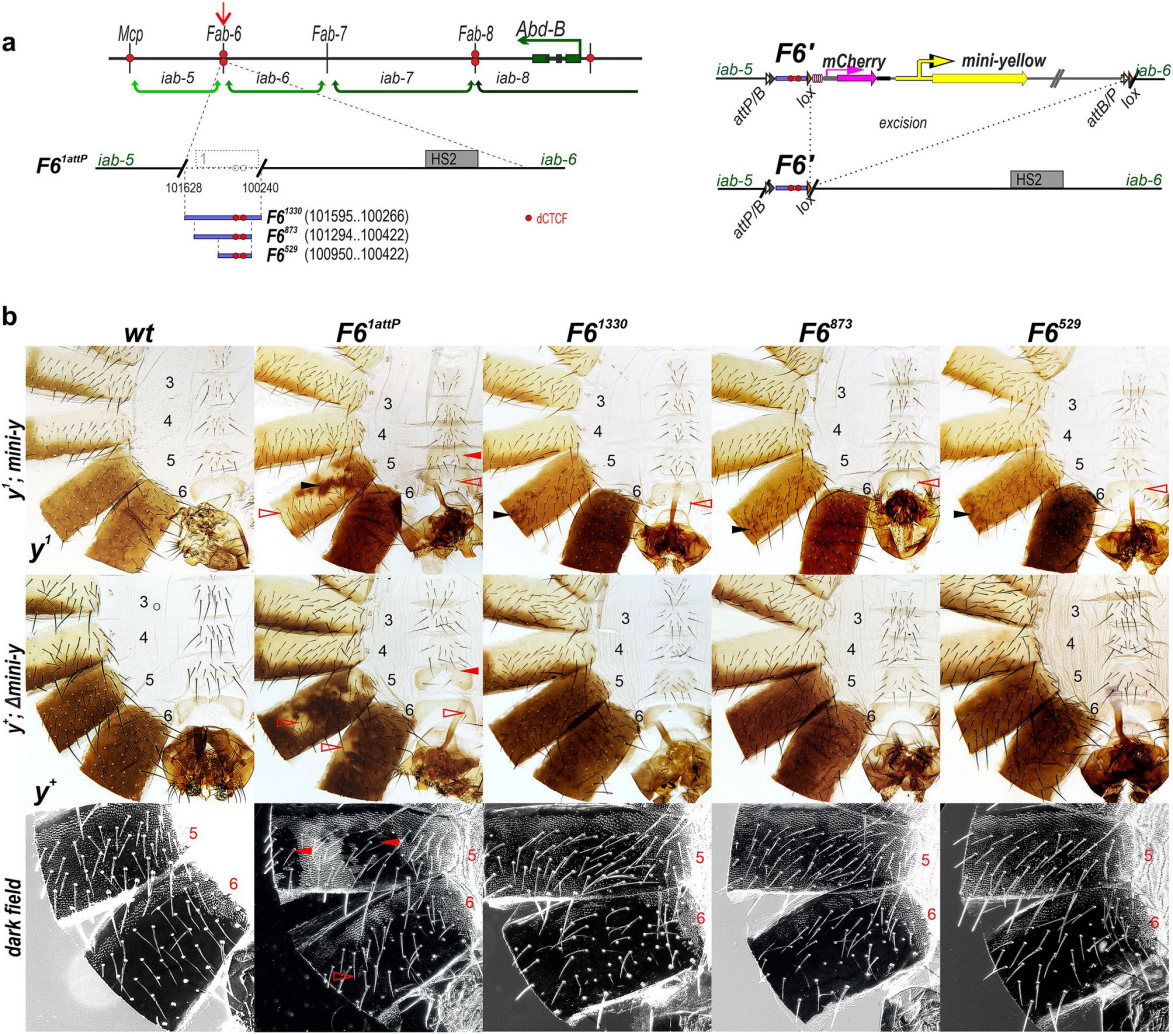
Mapping minimal sequences in HS1 *Fab-6* required for boundary function. (**a**) Schematic diagram of the *Fab-6* replacements**.** The coordinates of the *Fab-6* fragments (*F6′*) are according to the BX-C sequence^61^. On the right side: scheme of the replacements after integration in *F6*^*1attP*^ platform. The *mCherry* and *mini-y* genes are indicated by magenta and yellow arrows respectively. (**b**) Morphology of the male abdominal segments (numbered) in *F6*^*1attP*^, *F6*^*1330*^, *F6*^*873*^ and *F6*^*529*^. The filled red arrowheads show morphological features indicative of GOF transformations. The empty red arrowheads show LOF transformations. Black arrowheads indicate pigmented spots that are the result of ectopic activation of the *mini-y* reporter. All other designations are the same as described in Fig. 1.

To more precisely map the sequences required for boundary function, we used CRISPR/Cas9 to generate a 1389 bp deletion, *F6*^*1attP*^, that excises all of HS1 plus several hundred bp of surrounding DNA (Fig. 1 and S1). The CRISPR/Cas9 construct carries a *dsRed* reporter (*3* × *P3-DsRed*) to select for deletion events. Also included is an *attP* site that can be used for boundary replacement experiments^46^. In the adult cuticle the HS1 deletion, *F6*^*1attP*^, displays a strong GOF transformation of segment A5 towards A6. This transformation is most clearly evident in the ventral sternite. Instead of the characteristic quadrilateral shape (*wt*: Fig. 1), the A5 sternite has a banana shape like that normally observed in A6. It also differs from *wt* in that it lacks bristles. On the other hand, there are patches of depigmented tissue in the A5 tergite, which is indicative of a LOF transformation towards A4. Interestingly, we also observed weak LOF transformations in A6. These include small depigmented patches in the A6 tergite, and an occasional bristle in the A6 sternite (*F6*^*1attP*^, Fig. 2). The depigmentation seen in the tergite would be consistent with an A6 to A4 LOF transformation.

A similar, though not identical mixture of GOF/LOF cuticle phenotypes in A5 and a weak LOF phenotype in A6 were observed by Iampietro et al.^8^ in their *Fab-6*^*3*^ deletion that removes part of *iab-5* and most but not all of HS1 (Fig. 1a). One notable difference is that the GOF transformations of the sternite were much more modest in *Fab-6*^*3*^ than in *F6*^*1attP*^. Presumably these differences reflect the locations of the deletion breakpoints.

### The *Fab-6* HS1 cooperates with HS2 in blocking crosstalk between the *iab-5* and *iab-6* in vivo

To further define sequences important for *Fab-6* function we generated three replacements. The largest was 1330 bp and contained nearly the entire sequence deleted in *F6*^*1attP*^. The two other replacements were 873 bp and 529 bp (Fig. 2a). All three included the two CTCF sites. We introduced these replacements into *F6*^*1attP*^ using the φC31 *attP/attB* integration system^46^. To monitor blocking activity in the context of BX-C, the replacement included a minimal *yellow* reporter, *mini-y* (Fig. 2a). The reporter has a 340 bp *yellow* promoter linked to a *yellow* cDNA but lacks the wing, body and bristle enhancers of the endogenous *yellow* gene. As a result, *mini-y* expression depends upon enhancers in the neighborhood. The *mini-y* was placed relative to the test boundary sequences so that it is located in the *iab-6* regulatory domain. We also included a second reporter, an *mCherry* gene, in the replacement construct, which was found to be useless due to high background expression.

In order to recover insertion events and also to monitor blocking activity, we used a *y*^*1*^ genetic background. In flies carrying the null *y*^*1*^ allele, the *tan* gene is still expressed under the control of *Abd-B* and A5 and A6 have a light brown-yellow instead of black pigmentation^47^ (Fig. 2b, *wt* *y*^*1*^). When *mini-y* is introduced into the *F6*^*1attP*^ deletion without a boundary, reporter expression is driven by enhancers in the fused *iab-5:iab-6* domain. As can be seen by the dark pigmentation in the A5 and A6 tergites, the enhancers drive *mini-y* expression in both segments. However, as is observed when expression of the endogenous *y* gene is driven by *Abd-B* in the starting *F6*^*1attP*^ platform, there is “tan” pigmentation along the posterior margin of A5 as well as patches elsewhere in the tergite without pigmentation indicative of a LOF transformation of A5 into A4 (Fig. 2b). In these cells, the fused domain is shut down and *mini-y* (like *Abd-B*) is not expressed. A different pattern of *mini-y* expression is evident when the 1330 bp HS1 sequence is included in the replacement. In this case, *mini-y* is expressed at high levels throughout the A6 tergite, while expression is (with the exception of variable number of small darkly pigmented dots) absent in A5. Thus, the 1330 bp fragment effectively blocks crosstalk between *iab-5* and *iab-6*. This is also true for the two smaller replacements, 873 bp and 529 bp. Both eliminate *mini-y* expression in A5 as effectively as the larger fragment. The blocking activity of these replacements is also evident in the morphological phenotypes of A5 in both *y*^*1*^ and *y*^+^ flies. Like *wt* the A5 tergite is covered in trichome hairs, while the sternite has a quadrilateral shape with many bristles. These features indicate that even the smallest DNA sequence effectively blocks crosstalk between *iab-5* and *iab-6*. There was, however, one anomaly. In replacements carrying *mini-y* the A6 sternite has several bristles that are not seen in *wt*. However, this is likely due to promoter competition between *mini-y* and *Abd-B* for the *iab-6* enhancers as it is not observed in the replacements after the *mini-y* is excised (Fig. 2b).

### *Fab-6* HS1 cannot substitute for *Fab-7*

**Figure 3:**
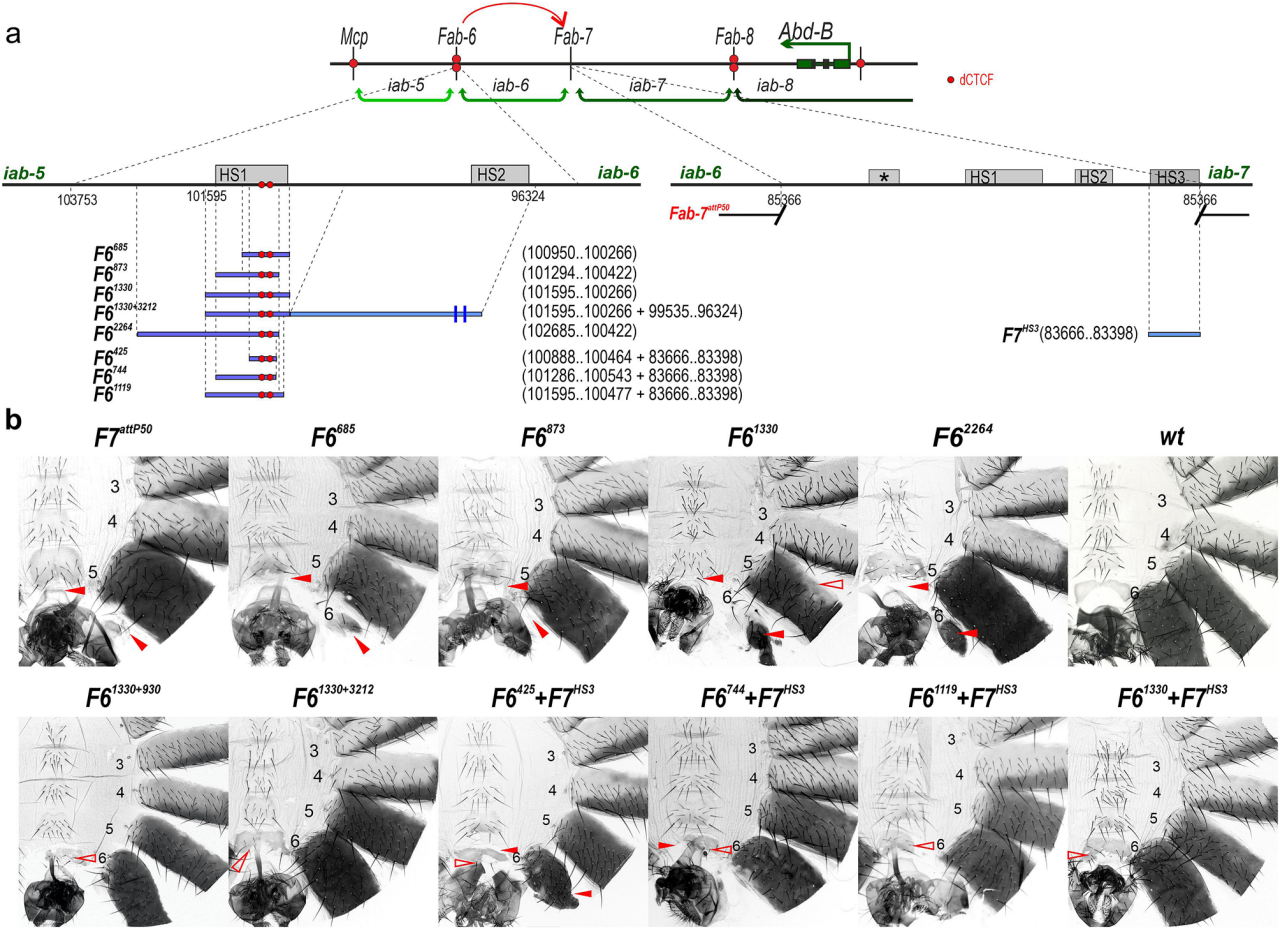
*Fab-6* HS1 cannot substitute for *Fab-7* unless it is combined with a PRE. (**a**) Maps of the *Fab-6* and *Fab-7* regions. The previously obtained *Fab-7*^*attP50*^ deletion^27^ is indicated by breaks in the black lines. (**b**) Morphology of the male abdominal segments in males with different *Fab-6* replacements. All other designations are the same as described in Figs. 1 and 2.

In previous studies a deletion *F7*^*attP50*^ (Fig. 3a) that replaces the four *Fab-7* nuclease hypersensitive regions with an *attP* site was generated^27^. We used this platform to further assess the functional properties of *Fab-6* HS1 (Fig. 3a). For this purpose, we introduced the 1330 bp and 873 bp *Fab-6* fragments described above, and a 685 bp fragment that has the same proximal end as the 529 bp fragment.

The *F7*^*attP50*^ deletion results in a complete GOF transformation, and in males not only A7 but also A6 are absent. Surprisingly, all three *Fab-6* HS1 fragments failed to rescue *F7*^*attP50*^ (Fig. 3b). In all three replacements, only a rudimentary A6 tergite is present, while there is no sternite. These findings indicate that *Fab-6* HS1 is not sufficient to reconstitute a functional boundary in a different BX-C chromosomal context.

One plausible explanation is that the two deletions we have used to test *Fab-6* HS1 boundary function are not equivalent. The *Fab-7* deletion removes all of the nuclease hypersensitive regions including the HS3, which has both boundary and PRE activity. By contrast, the *Fab-6* deletion only removes HS1 but not the HS2 PRE. If this explanation is correct, then it may be possible to reconstitute *Fab-7* by combining DNA fragments that encompass *Fab-6* HS1 and HS2.

To test this possibility, we generated two *Fab-7* replacements. The first, *F6*^*1330*+*3212*^, has HS1 plus a large fragment extending to either side of HS2. The second, *F6*^*1330*+*930*^*,* has a smaller HS2 fragment. Figure 3 shows that for both of these replacements, the A6 segment is almost wild type, indicating that they are effective substitutes for *Fab-7*. In both replacements the tergite is fully formed and the trichome hairs are largely restricted to the anterior and lateral edges as in *wt*, while the sternite has the appropriate banana shape. However, for both replacements there are two anomalies: there are patches of ectopic trichomes on the A6 tergite, while the sternite has several bristles. These weak LOF defects would suggest that the boundary bypass activity of both *F6*^*1330*+*3212*^ and *F6*^*1330*+*930*^ is not fully effective. Alternatively, since both HS1 and HS2 have PRE activity, the two together could sometimes silence *iab-6*.

We also tested a 2264 bp fragment that includes the 1339 bp HS1, but extends in the opposite direction towards *iab-5*. Unlike *F6*^*1330*+*3212*^ or *F6*^*1330*+*930*^, *F6*^*2264*^ failed to substitute for *Fab-7*, suggesting that there are no additional sequences conferring insulator function on the centromere proximal side of *Fab-6* HS1.

### Combination of *Fab-6* HS1 and *Fab-7* HS3 substitutes for *Fab-7*

The finding that *Fab-6* HS1 substitutes for *Fab-7* when combined with HS2 suggests that that HS2 has both PRE and boundary activity like *Fab-7* HS3^39^. If this idea is correct, then *Fab-6* HS1 might be able to substitute for *Fab-7* when linked to *Fab-7* HS3. To explore this idea, we combined four different *Fab-6* HS1 fragments (Fig. 3b and Fig. S2) with *Fab-7* HS3. The largest was *F6*^*1330*^, while the smallest was *F6*^*425*^^45^. We also tested two intermediate *Fab-6* fragments, *F6*^*744*^ and *F6*^*1119*^ (Fig. 3).

The two larger combinations, *F6*^*1330*^ + *F7*^*HS3*^ and *F6*^*1119*^ + *F7*^*HS3*^, have similar activities. In both cases they rescue the GOF transformations of the *F7*^*attP50*^ deletion. The sternite has the appropriate banana shape, while the tergite is *wt* in size (Fig. 3b). However, as observed for *F6*^*1330*+*3212*^, the sternite usually has a few bristles, while there are ectopic trichome patches on the tergite. Surprisingly, the two smaller combinations, *F6*^*744*^ + *F7*^*HS3*^ and *F6*^*425*^ + *F7*^*HS3*^, fail to fully rescue *F7*^*attP50*^. The GOF phenotypes are most pronounced in the *F6*^*425*^ + *F7*^*HS3*^ combination where both the sternites and tergites are significantly reduced in size. In the case of *F6*^*744*^ + *F7*^*HS3*^ the sternites are typically misshapen while the tegrites have nicks or are smaller than normal. As was the case for the larger *F6* fragments, weak LOF phenotypes are also observed, likely due to minor defects in bypass activity.

### *Fab-6* HS1 + HS2 deletions have a more complete GOF phenotype

Our *Fab-7* replacement experiments indicate that *F6*^*1330*^ (HS1) must be combined either with the *Fab-6* HS2 or *Fab-7* HS3 to rescue the *F7*^*attP50*^ deletion. To further pursue the role of the *Fab-6* HS2, we designed a CRISPR/Cas9 deletion, *F6*^*1*+*2attP*^, which is 5995 bp and removes both HS1 and HS2.

As anticipated from studies on *Fab-7*, the phenotype of *F6*^*1*+*2attP*^ differs from *F6*^*1attP*^. As shown in Fig. 1a, there is a nearly complete GOF transformation of A5 towards A6. The difference in the phenotypic effects of *F6*^*1*+*2attP*^ and *F6*^*1attP*^ are most clearly evident in the A5 tergite. While the A5 tergite in *F6*^*1attP*^ has patches of unpigmented cuticle indicative of an LOF transformation, the A5 tergite in *F6*^*1*+*2attP*^ is fully pigmented in > 90% of the males. Similarly, though the A5 sternite in *F6*^*1attP*^ has an A6-like banana shape, it also has several bristles, which are not present in the A6 sternite (Fig. S3). In *F6*^*1*+*2attP*^ the GOF transformation is more complete as the sternite lacks bristles in 70–80% of the males.

Thus, as was observed for *Fab-7* deletions which retain or remove HS3^33^, removing both *Fab-6* HS1 and HS2 results in much more complete GOF transformation than HS1 only. In this context, we would note that a strong GOF transformation of A5 was not observed by Iampietro et al.^8^ in the *Fab-6*^*2*^ deletion (Fig. 1b) which removes both *Fab-6* HS1 and HS2. Instead, they observed a mixed GOF/LOF phenotype not altogether different from their smaller deletion, *Fab-6*^*3*^. We suspect that the difference in phenotypes is that their deletion removes a larger segment from *iab-5*.

### HS2 contributes to *Fab-6* boundary activity

**Figure 4:**
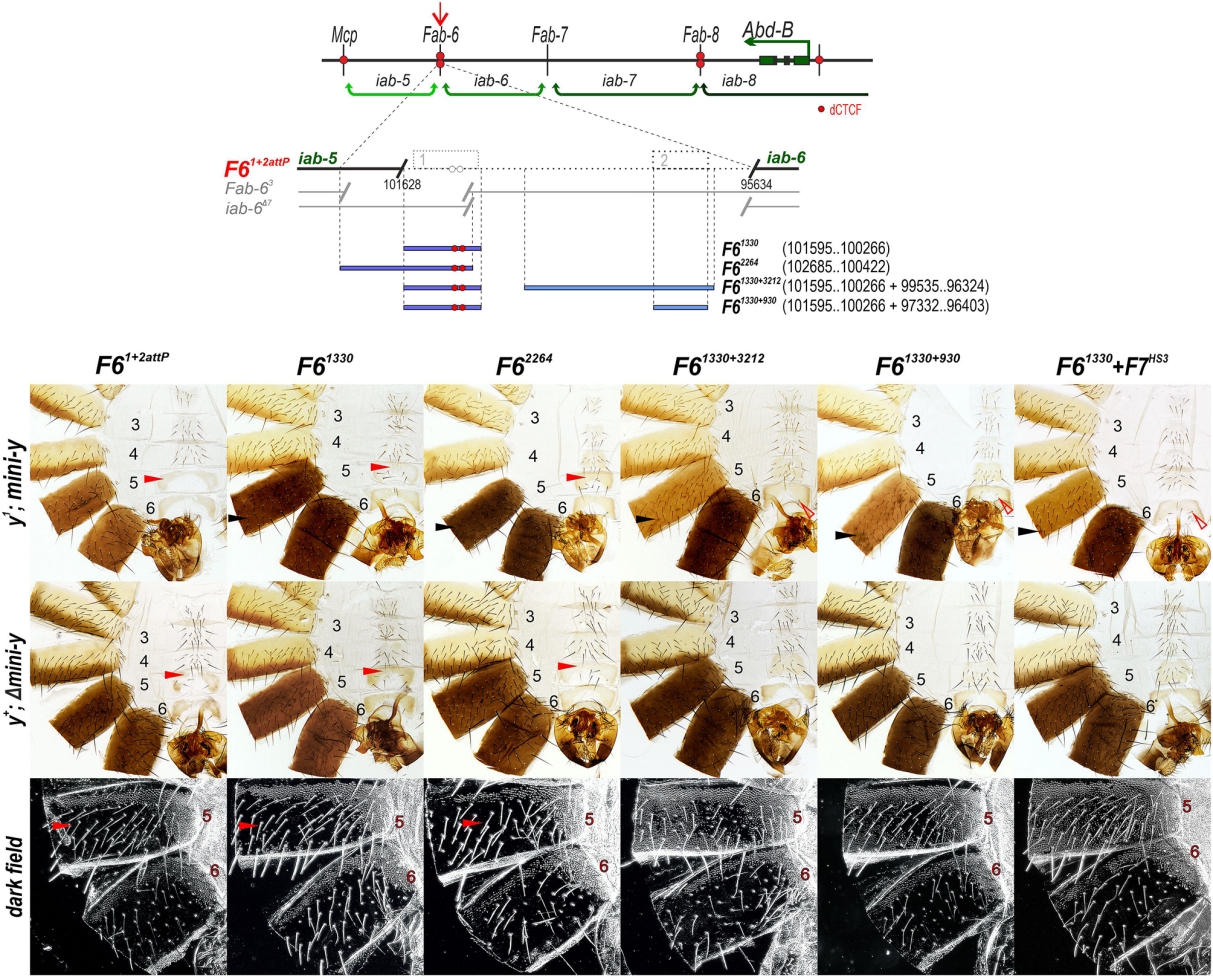
The *Fab-6* HS2 cooperates with HS1 to reconstitute a fully functional boundary. On the top: schematic diagram of the *Fab-6* region, the *F6*^*1*+*2attP*^ deletion and the different DNA fragments tested for boundary activity. Morphology of the male abdominal segments in males carrying different *Fab-6* fragments in *F6*^*1*+*2attP*^. All other designations are the same as described in Figs. 1 and 2.

Next, we generated four *F6*^*1*+*2attP*^ replacements. The first replacement, *F6*^*1330*^, has only HS1. The second, *F6*^*1330*+*3212*^, has both HS1 and HS2, but lacks 730 bp between the HS1 and HS2. The third, *F6*^*1330*+*930*^, has only HS1 and HS2. The fourth replacement was *F6*^*2264*^ which contains the 1330 bp HS1 sequence, but extends into *iab-5* (see above). These fragments were introduced into the *mini-y* replacement vector (Fig. 4).

Figure 4 shows that the two replacements that contain only HS1, *F6*^*1330*^ and *F6*^*2264*^ are unable to insulate *mini-y* and it is expressed in A5 and A6. Consistent with ineffective insulation, the sternite in A5 has a banana shape while the arrangement of the trichomes in the A5 tergite resembles that normally observed in A6 (they are restricted to the anterior and dorsal lateral margins). Both of these morphological features are indicative of a GOF transformation of A5 towards A6 and are observed with and without the *mini-y* reporter. It is worth noting that the GOF transformations are less complete when the *mini-y* reporter is present. Though the A5 sternite has a banana shape, it has some bristles while there are small patches of trichomes on the tergite. However, the ectopic bristles and trichomes disappear and the flies resemble the starting deletion, *F6*^*1*+*2attP*^ after excision of *mini-y*.

A different result is obtained when the replacement combined *Fab-6* HS1 plus HS2. The pigmentation pattern in the A5 and A6 tergites of *y*^*1*^; *F6*^*1330*+*3212*^ *mini-y* and *y*^*1*^; *F6*^*1330*+*930*^ *mini-y* indicates that the *mini-y* is (with the exception of a few small dots of pigmented cuticle in A5: see arrowhead) insulated from enhancers in *iab-5*. The morphology of the dorsal and ventral cuticle in A5 is also normal. The one possible exception is some gaps in the trichome field in the smaller *F6*^*1330*+*930*^ replacement.

These findings suggest that *iab-6* PRE, HS2, contributes to boundary function much like is observed for *Fab-7* where HS3 functions both as the *iab-7* PRE and as part of the *Fab-7* boundary. If this suggestion is correct, then one would predict that *Fab-7* HS3 would be able to substitute for *Fab-6* HS2. To test this prediction, we combined *Fab-6* HS1 with *Fab-7* HS3 (*F6*^*1330*^ + *F7*^*HS3*^). As shown in Fig. 4, the *F6*^*1330*^ + *F7*^*HS3*^ combination also restores boundary function.

## Discussion

Parasegment/segment identity depends upon the proper functioning of the different elements in each BX-C regulatory domains^3^. These elements include parasegment specific initiators that are determine the *on/off* state of domains early in development, elements that function to maintain the *on/off* state (PREs/TREs), and enhancers that drive expression of the homeotic genes in patterns appropriate for each parasegment. The regulatory domains are bracketed by chromatin boundaries, which are responsible for insulating regulatory elements within one domain from interactions with regulatory elements in neighboring domains. In addition to insulating activity, some of the BX-C boundaries can mediate interactions between enhancers in the regulatory domains and the relevant target promoter^25,26,34^. This boundary bypass function requires long distance physical interactions between boundaries and compatible architectural elements associated with the promoters of the three homeotic genes.

Here we have investigated the insulating and bypass activities of the *Fab-6* boundary, which is located between *iab-5* and *iab-6*. Though the sequence organization of *Fab-6* is very different from that of the *Fab-7*, there are some parallels in their properties. Deletions that remove *Fab-7* HS* + HS1 + HS2 but retain the HS3 *iab-7* PRE result in a mixed GOF/LOF transformation of A6^33^. A similar mixed GOF/LOF phenotype, in this case in A5 is observed in deletions that remove only *Fab-6* HS1. For the *Fab-6* HS1 deletion, the GOF phenotype results from activation of *iab-6* in PS10 by the *iab-5* initiator, while the LOF phenotype is due to the silencing of *iab-5* and *iab-6* in PS10 by a mechanism dependent on the *iab-6* PRE. *Fab-7* deletions that remove all HS sites result in a complete GOF transformation of A6 (PS11) into A7 (PS12)^33^. A similar strong GOF transformation is observed when both *Fab-6* HS1 and HS2 are deleted.

Also, in both cases boundary function is supplemented by elements that have PRE activity. For *Fab-7*, the *iab-7* PRE (HS3) contributes to its boundary function. *Fab-6* also depends on elements that have boundary and PRE activity. In transgene studies Perez-Lluch et al.^41^ found that *Fab-6* HS1 and HS2 silenced a *white* reporter by PcG dependent mechanism. In addition, HS1 also functions as a boundary in an enhancer blocking assays, and this activity depends upon the chromosomal architectural protein dCTCF. The conclusion that HS1 has boundary activity is supported by the studies of Iampietro et al.^8^ as well as our experiments showing that the *F6*^*529*^ fragment containing CTCF sites can rescue the GOF/LOF transformations of a *F6*^*1attP*^ deletion. On the other hand, even larger fragments extending to either side of the *Fab-6* HS1 region are unable to rescue *Fab-7*^*attP50*^ and *F6*^*1*+*2attP*^ deletions. However, *Fab-6* HS1 can substitute for *Fab-7* when it is combined with HS2. This observation suggests that HS2 functions not only as a PRE silencer, but also as a boundary. Consistent with this idea, we found that the *Fab-7* deletion, *F7*^*attP50*^, can also be almost fully rescued by combining *Fab-6* HS1 and *Fab-7* HS3. Rescuing of the large *Fab-6* HS1 + HS2 deletion also requires a combination of *Fab-6* HS1 with either HS2 or *Fab-7* HS3. In this respect, it is interesting to note that genome-wide studies on *Drosophila* TADs (topologically associated domains) suggest that PREs are involved in the formation of chromatin loops^48,49^. It is not clear at this point to what extent this architectural function is due to the PcG silencing activity of these elements as opposed to a distinct boundary-like function.

**Figure 5:**
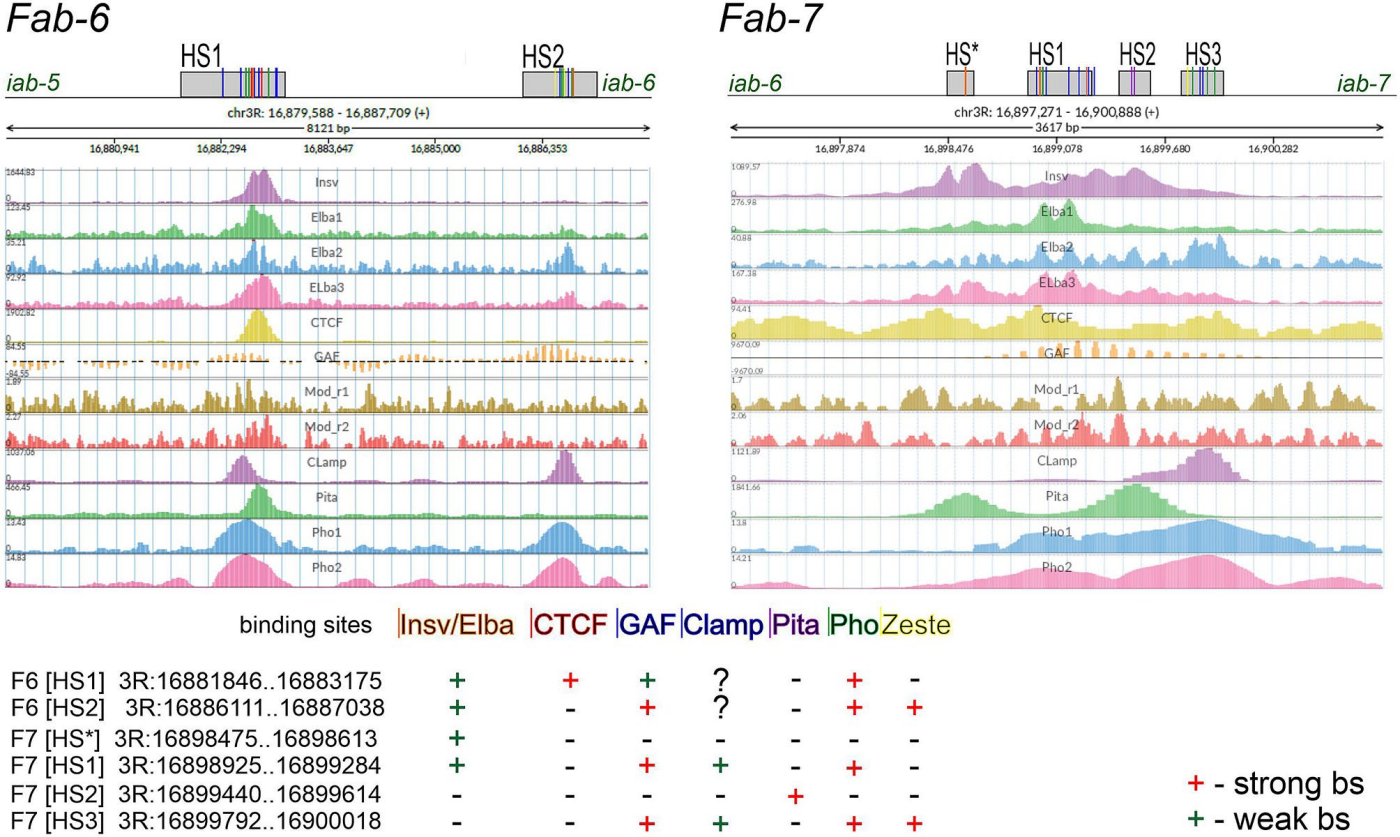
Comparison of chromatin protein binding to the *Fab-6* and *Fab-7* regions. The data are from a public functional genomics data repository GEO. The DNAse I hypersensitive sites (HS) of *Fab-6* and *Fab-7* are indicated as grey boxes. Binding sites for GAF, Clamp, INSV/ELBA complex, PHO, Zeste, Pita, dCTCF are indicated by short lines that correspond to the color of the protein names.

It is interesting to compare *Fab-6* with the two other *Abd-B* boundaries, *Fab-7* and *Fab-8*. Like *Fab-8*, there are two CTCF motifs in *Fab-6* HS1 that bind dCTCF in ChIP experiments^43,44,50^. In spite of this similarity, *Fab-6* HS1 is clearly not equivalent to *Fab-8*. Unlike *Fab-6* HS1, a 337 bp *Fab-8* fragment that includes the two CTCF sites can fully substitute for *Fab-7*^34,40^. While dCTCF does not bind directly to *Fab-7*, ChIP-seq analysis^51–54^ indicate that several chromosomal architectural factors known to be important for *Fab-7* boundary functions also appear to be associated with *Fab-6* HS1. These common factors include the BEN DNA binding domain factors, Insensitive and Elba, Pita, and several proteins (GAF, Mod(mdg4) and CLAMP) that are thought to be components of the LBC (Late Boundary Complex) (Fig. 5). All of these factors also have recognition sequences in *Fab-7* HS*, HS1 or HS2^25,27,55–57^ and are known to play a role in their boundary functions. As for *Fab-6* HS2, ChIP experiments indicate that the Polycomb DNA binding protein Pleiohomeotic (Pho), and the three LBC proteins GAF, Mod(mdg4) and CLAMP localize to this hypersensitive region. The same proteins are found associated with *Fab-7* HS3. These similarities in protein composition would potentially explain why *Fab-7* HS3 can substitute for *Fab-6* HS2.

Our experiments highlight important differences in the properties of *Drosophila* boundary elements from those that have been reported for boundaries in mammals. All that is thought to be needed for full boundary function in mammals is the binding of a single protein, CTCF, to its 15 bp recognition sequence^58,59^. While *Fab-6* has two dCTCF sites, these two sites are clearly not sufficient for boundary function in the context of BX-C. Instead, boundary activity requires two DNA sequences of ~ 1 kb in length and ChIP experiments indicate that at least eight different DNA binding proteins are associated with *Fab-6* HS1 and HS2 in vivo (Fig. 5). Given that ChIPs have been done for only a small fraction of the predicted *Drosophila* DNA binding proteins^60^, it reasonable to think that many more proteins will interact with HS1 and HS2 and contribute to their boundary functions. Further analysis of the proteins associated with *Fab-6* HS1 and HS2, and mutational studies will be required to confirm and extend these suggestions.

## Methods

### Generation of *F6*^*1attP*^ and *F6*^*1*+*2attP*^ deletions

The deletions were obtained by CRISPR/Cas9-induced homologous recombination. As a reporter, we used *pHD-DsRed* vector that was a gift from Kate O’Connor-Giles (Addgene plasmid # 51434). The plasmid was constructed in the following order: proximal arm-*3* × *P3:DsRed-distal* *arm*. Arms for homology recombination were amplified by PCR from DNA isolated from *Oregon* line. For generation of the *F6*^*1attP*^ deletion, homology arms were obtained by DNA amplification between primers: F6ProxDRI: TATGAATTCCCCGAGACTAAACATAATTCGC; F6ProxRNde: TATCATATGACTGGCACCAGCTAATTGACAA; F6DistDSpe: TTACTAGTCATATTTGGGGATTTCTCTAAGTTTG; F6DistRPsc.

TTTACATGTCCGTGGTCGTTTTTTGTGGTT. For generation of the *F6*^*1*+*2attP*^ deletion only distal arm was changed: i6SGII: ATTAGATCTGCAAACTCAGTGGGCTTTTC; i6SXho: ATTCTCGAGCTGGTTGTTGGGATCGGG. The guide RNAs were selected using the program “CRISPR optimal target finder” (O’Connor-Giles Lab): for *F6*^*1attP*^ deletion - GTGCGCTAAGCACGCATATT and GTGTGTGGTCCGCAATACAG, for *F6*^*1*+*2attP*^ - AGTTTGCAAAGACAGTCCGT and GTGTGTGGTCCGCAATACAG. The breakpoints of the designed deletions: *F6*^*1attP*^ - 3R:16883201..16881813 and *F6*^*1*+*2attP*^ - 3R:16887807..16881813 16,869,768 according Genome Release r6.36.

To generate the desired deletions, the plasmid construct was injected into *58492* (Bloomington Drosophila Stock Center) embryos together with two gRNAs. The F0 progeny was crossed with *y* *w*; *TM6/MKRS* flies. Flies with potential deletions were selected on the basis of dsRed-signal in eyes and the posterior part of their abdomens and these flies were crossed with *y* *w*; *TM6/MKRS* flies. All independently obtained flies with dsRed reporter were tested by PCR. The successful deletion events were confirmed by sequencing of PCR products.

### Generation of transgenic lines carrying different insertions in the *Fab-7*^*attP50*^, *F6*^*1attP*^ and *F6*^*1*+*2attP*^ landing platforms

The *Fab-7*^*attP5*0^ landing platform was described previously^27^. The test replacement fragments were inserted in a plasmid carrying the *rosy* reporter and an *attP* site described in^27^.

For the *F6*^*1attP*^ and *F6*^*1*+*2attP*^ landing platforms the replacement vector was a plasmid with the *mini-yellow* and *mCherry* reporter as shown in Fig. S1. The *Fab-6* fragments were obtained by PCR amplification. Their coordinates are shown in Figures according to the published sequences of the Bithorax complex^61^.

Integration of the plasmids in the landing platforms was achieved by injecting the plasmid and a vector expressing the *фC31* recombinase into embryos of *yw;* *F6*^*1attP*^*/* *F6*^*1attP*^ or *yw;* *F6*^*1*+*2attP*^/*F6*^*1*+*2attP*^ line. The successful integrations were selected on the basis of expression of *yellow* reporter in abdominal segments. The integration of the replacement DNA fragments was confirmed by sequencing of PCR fragments.

The *yellow* and *mCherry* reporters were excised by Cre-mediated recombination between the *lox* sites.

All stocks are available upon request.

### Cuticle preparations

Cuticle preparations were carried out as described by^26^.

## Supplementary Information

Supplementary Information
