# Supplementary material for: Mapping of functional elements of the Fab-6 boundary involved in the regulation of the Abd-B hox gene in Drosophilamelanogaster
Source: Sci Rep. 2021 Feb 18;11:4156. doi: 10.1038/s41598-021-83734-8 (PMC7892861; doi:10.1038/s41598-021-83734-8)
Supplement: Supplementary file 1 — Supplementary Information [file 41598_2021_83734_MOESM1_ESM.pdf]

Electronic Supplementary Material to

Mapping of functional elements of the *Fab-6* boundary involved in the regulation of the *Abd-B* hox gene in *Drosophila melanogaster*

Nikolay Postika[1], Paul Schedl[2,3], Pavel Georgiev,[1*] Olga Kyrchanova[1*]

[1]Department of the Control of Genetic Processes, Institute of Gene Biology Russian Academy of Sciences, 34/5 Vavilov St., Moscow 119334, Russia;

[2]Laboratory of Gene Expression Regulation in Development, Institute of Gene Biology Russian Academy of Sciences, 34/5 Vavilov St., Moscow 119334, Russia;

[3]Department of Molecular Biology, Princeton University, Princeton, NJ, 08544, USA.

*Corresponding author:

E-mail: olgina73@gmail.com (OK)

E-mail: georgiev_p@mail.ru (PG)

Supplementary Information

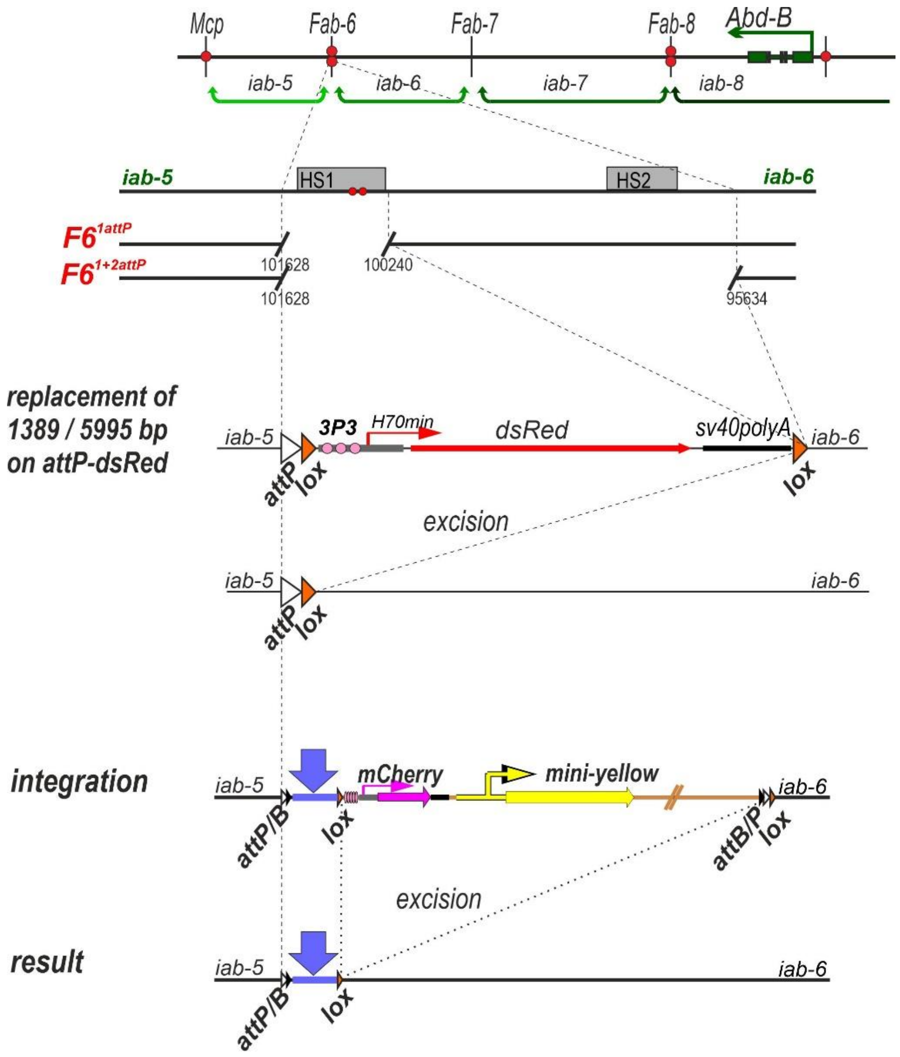

Figure S1. Strategy for generating the *F6*^{1attP} *and F6*$^{1+2attP}$ replacement lines. Scheme of the regulatory regions associated with the *Abd-B* gene. The *Abd-B* promoter and direction of its transcription are indicated by green arrow. DNAase 1 hypersensitive regions, HS1 and HS2, are indicated as gray boxes. The *F6*^{1attP} *and F6*$^{1+2attP}$ deletions are indicated by breaks in the black lines. The coordinates of the endpoints are according to the complete sequence of BX-C in SEQ89E numbering. The *F6*^{1attP} *and F6*$^{1+2attP}$ deletions were obtained by substituting the designated DNA sequences with an *attP* site (white triangle) and the *dsRed* marker gene (thick red arrow) with SV40 terminator at the 5'-end (black srripe), flanked by *lox* sites (orange triangle). During the final step, the *dsRed* gene was deleted by recombination between the *lox* sites. The plasmid that was injected into the *F6*^{1attP} or *F6*$^{1+2attP}$ lines contains the *attB* site (black triangle) for integration and the *lox* site for the excision of the *yellow* (thick yellow arrow) and *mCherry* (thick magenta arrow) reporters. Both reporters and plasmid body (brown stripes) were excised by Cre-mediated recombination between the *lox* sites. As a result, the test replacement fragments were inserted in place of the deletion.

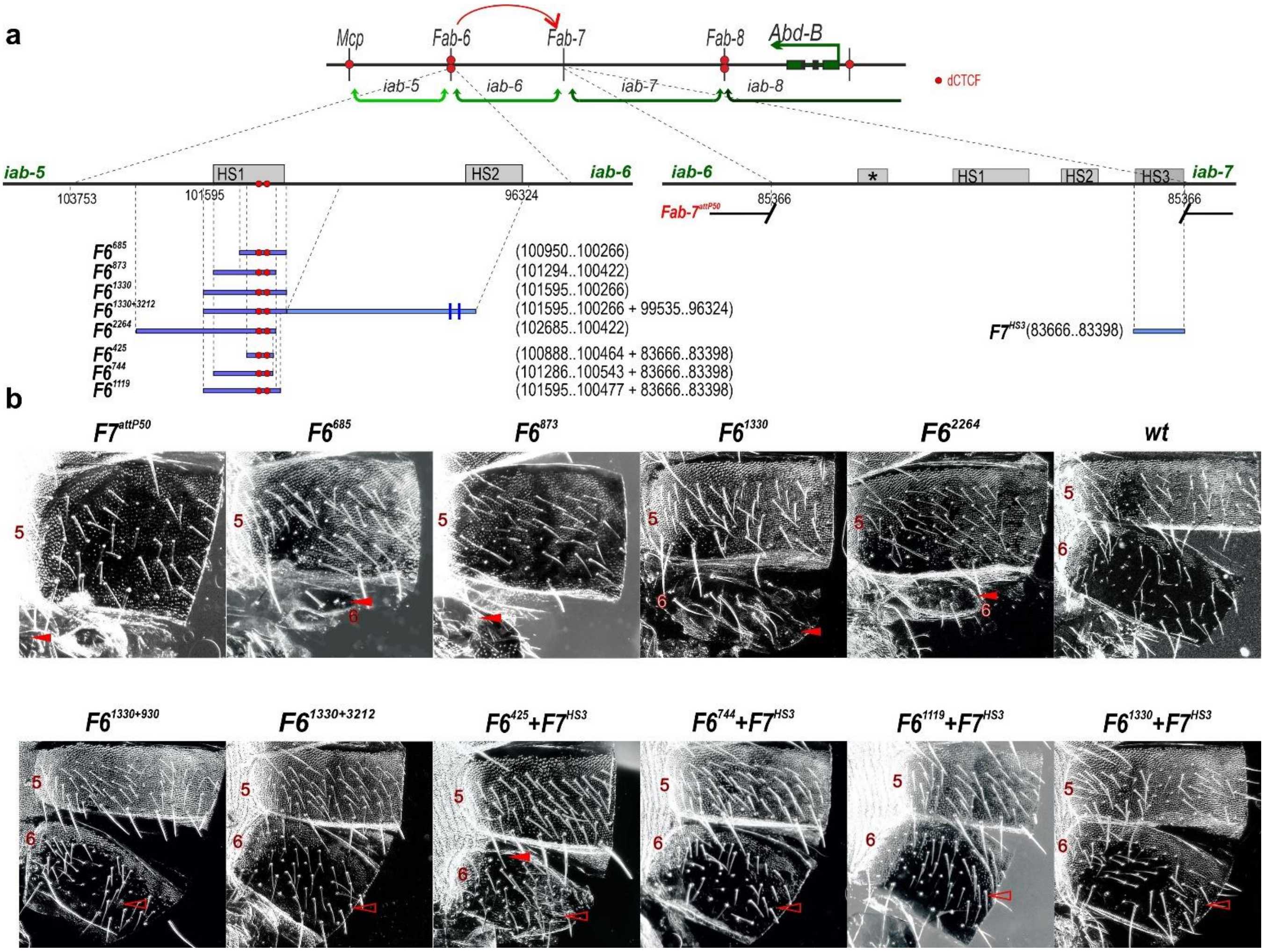

Figure S2. The PRE is critical to the ability of *Fab-6* HS1 to replace *Fab-7* boundary. (A) Maps of the *Fab-6* and *Fab-7* regions. Hypersensitive regions are indicated by gray boxes. The previously obtained *Fab-7*^{attP50} deletion[27] is indicated by breaks in the black lines. (B) Morphology of the 5th and 6th male abdominal segments in dark field in males with different *Fab-6* replacements. The filled red arrowheads show morphological features indicative of GOF transformations. The empty red arrowheads show the signs of the LOF transformation. (C) Morphology of the male abdominal segments in dark field in males with combination of different *Fab-6* HS1 and the *iab-7* PRE, HS3. All other designations are the same as described in Fig. 1 and 2.

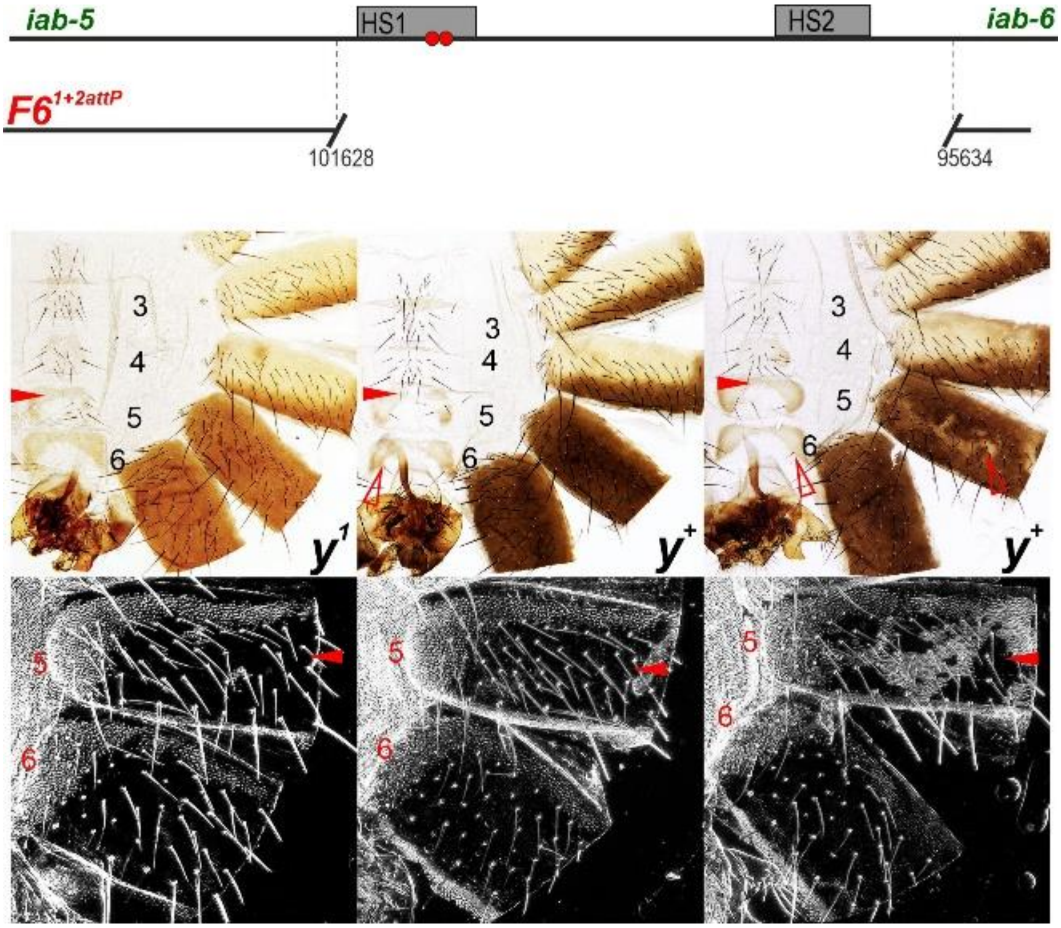

Figure S3. Variants of the *F6*$^{1+2attP}$ males A5 and A6 morphology. Scheme of the *F6*$^{1+2attP}$ deletion. HS1 and HS2 are shown as gray boxes. Morphology of the *F6*$^{1+2attP}$ male's abdominal segments.
